# An ultrasensitive hybridization chain reaction-amplified CRISPR-Cas12a aptasensor for extracellular vesicle surface protein quantification

**DOI:** 10.7150/thno.49047

**Published:** 2020-08-13

**Authors:** Shan Xing, Zedong Lu, Qi Huang, Huilan Li, Yu Wang, Yanzhen Lai, Yi He, Min Deng, Wanli Liu

**Affiliations:** 1 Department of Clinical Laboratory, State Key Laboratory of Oncology in South China, Collaborative Innovation Center for Cancer Medicine, Guangdong Key Laboratory of Nasopharyngeal Carcinoma Diagnosis and Therapy, Sun Yat-sen University Cancer Center, 651 Dongfeng Road East, Guangzhou 510060, P. R. China.; 2 School of Biomedical Engineering, Sun Yat-sen University, No. 132 Waihuandong Road, University Town, Guangzhou 510006, PR China.; 3 Heyuan People's Hospital, Heyuan, China.; 4 Affiliated Cancer Hospital & Institute of Guangzhou Medical University, No.78, Hengzhigang Road, Guangzhou 510095, P. R. China.

**Keywords:** Tumor-derived exosomes, CRISPR-Cas12a, Hybridization chain reaction, Aptamer, Fluorescence

## Abstract

Tumor-derived extracellular vesicle (TEV) protein biomarkers facilitate cancer diagnosis and prognostic evaluations. However, the lack of reliable and convenient quantitative methods for evaluating TEV proteins prevents their clinical application.

**Methods:** Here, based on dual amplification of hybridization chain reaction (HCR) and CRISPR-Cas12a, we developed the apta-HCR-CRISPR assay for direct high-sensitivity detection of TEV proteins. The TEV protein-targeted aptamer was amplified by HCR to produce a long-repeated sequence comprising multiple CRISPR RNA (crRNA) targetable barcodes, and the signals were further amplified by CRISPR-Cas12a collateral cleavage activities, resulting in a fluorescence signal.

**Results:** The established strategy was verified by detecting the TEV protein markers nucleolin and programmed death ligand 1 (PD-L1). Both achieved limit of detection (LOD) values as low as 10^2^ particles/µL, which is at least 10^4^-fold more sensitive than aptamer-ELISA and 10^2^-fold more sensitive than apta-HCR-ELISA. We directly applied our assay to a clinical analysis of circulating TEVs from 50 µL of serum, revealing potential applications of nucleolin^+^ TEVs for nasopharyngeal carcinoma cancer (NPC) diagnosis and PD-L1^+^ TEVs for therapeutic monitoring.

**Conclusion:** The platform was simple and easy to operate, and this approach should be useful for the highly sensitive and versatile quantification of TEV proteins in clinical samples.

## Introduction

Extracellular vesicles (EVs) are a heterogeneous class of membrane-bound phospholipid vesicles that are shed by cells into a variety of bodily fluids 1, 2. The CD9, CD63 and CD81 tetraspanin proteins serve as putative common EV markers and have been routinely applied in the immuno-isolation of circulating EVs 3-7. Tumor-derived EV (TEV) protein biomarkers are closely associated with the diagnosis, prognosis, progression and immune response of certain cancers 7-12; however, they are often expressed at extremely low levels and thus require a highly sensitive detection method 13. Conventional EV detection methods such as ELISA, Western blotting or flow cytometry molecular characterization generally require large amounts of biopsy samples (> 1 mL) for sufficient sensitivity and time-consuming ultracentrifugation for purification and concentration 14, 15. Despite the improved sensitivity and efficiency, recent developed biosensors, such as the thermophoretic aptasensor 16, nanoplasmonic exosome sensors 17, ExoPCD-chips 18, integrated magnetic-electrochemical exosome sensors 19, and micro-nuclear magnetic resonance 20, often require expensive equipment or sophisticated sensing procedures, creating critical challenges for clinical applications. We previously established a proximity ligation assay-recombinase polymerase amplification (PLA-RPA) transcription-mediated amplification (TMA) assay 21 to detect trace TEV protein directly in serum. This platform is highly sensitive and requires a less complex instrument; however, possible issues limiting its wide clinical application include a high cost, background noise, stringent reaction conditions for RPA and the laborious process of labeling oligonucleotides to antibodies. Therefore, clinically feasible techniques to detect low-abundance EV proteins that are sensitive, easy to operate and cost-effective urgently need to be developed to expand the routine clinical utility of circulating TEVs.

The hybridization chain reaction (HCR) is a simple but robust and efficient isothermal amplifycation process 22, 23. Due to its enzyme-free reaction nature, HCR is low cost and easy to perform 24. Moreover, the HCR isothermal amplification technique is a probe amplification technique that does not involve target amplification, effectively reducing cross-contamination from amplicons and false-positive results, which often occur in RPA and PCR 24. Beyond these properties, HCR has the advantage of structural versatility, enabling DNA to be used as both a recognition molecule (through combination with antibodies or aptamers) and an amplifying transducer for biosensing simultaneously 25. Given these favorable attributes, the application of HCR as a biosensor has undergone tremendous development during the last few decades 26-28.

Clustered regularly interspaced short palindromic repeats (CRISPR)/Cas12a has recently been demonstrated to cleave the nonspecific single-stranded DNA (ssDNA) reporter induced by Cas-CRISPR RNA (crRNA) target recognition-and-cleavage events 29, 30. Cas endonuclease activity is activated only when the target DNA has the perfect complementary sequence to the crRNA and is thus highly specific 31-35. The method is also highly efficient, providing collateral cleavage rates of at least 1250 turnovers/second per target DNA recognition 29. Coupled with nucleic acid amplification technique, CRISPR/Cas12a enables DNA detection with sensitivity at the attomolar level 29, 36, 37. Despite the highly efficient signal amplification ability, most of the CRISPR-based sensors reported to date are employed in nucleic acid detection 38-43. Nucleic acid aptamers obtained by *in vitro* selection methods 44-47 generalize CRISPR-based sensor application to a wide range of non-nucleic acid targets such as ions and small molecules; however, access to protein detection remains limited 48-50.

Aptamers are a low-cost alternative to antibodies but exhibit comparable excellent affinities and specificities towards target proteins 44-46. AS1411 (a 26-mer G-rich DNA aptamer) has been widely proven to bind to nucleolin protein with high affinity and has even been assessed in oncology clinical trials 51. Notably, nucleolin protein is overexpressed on the surfaces of most malignant cells and malignant cell-derived exosomes but not on normal cells 52, 53. Owing to these two features, we initially set up our model for nucleolin^+^ TEV detection. Given that circulating EV programmed death ligand 1 (PD-L1) has been reported to be an ideal predictive biomarker for monitoring clinical responses to anti-PD-1/PD-L1 therapy 9 and that an aptamer for this protein has been reported previously 54, we also explored the application of our platform for PD-L1^+^ TEV detection.

Here, we combined aptamers that specifically bind to protein targets on EVs to transduce the target recognition event signal to DNA effectors, HCR for DNA amplification and the production of multi-repeated CRISPR-targetable DNA units, and CRISPR/Cas12a collateral endonuclease activity for amplification, real-time DNA detection and readable signal outputs to develop an alternative CRISPR Cas12a strategy, termed the apta-HCR-CRISPR assay, to detect nucleolin^+^ and PD-L1^+^ TEVs. Based on the concept of apta-HCR-CRISPR, we developed our sensitive, simple, clinically feasible and cost-effective platform as a potential broad biosensing strategy for the detection of EV proteins.

## Methods

### Chemicals

The oligonucleotides used were synthesized and purified by polyacrylamide gel electrophoresis (PAGE) or HPLC by Sangon Biotechnology Co., Ltd. (Shanghai, China) or Ruibiotech Co. (Guangzhou, China). All of the DNA sequences and modifications are listed in Table S1. Exosome-human isolation reagent (anti-CD63, anti-CD9 and anti-CD81 antibody-conjugated magnetic beads (MBs)) and 10× phosphate-buffered saline (PBS) (0.1 M, pH 7.4) were purchased from Thermo Fisher Scientific (Rockford, IL, USA). Streptavidin-coated MBs were acquired from Roche, Basel, Switzerland. EV-free fetal bovine serum (FBS) was obtained from Suer, Shanghai, China. The EV identification antibodies used were as follows: anti-CD9 (Proteintech, 20597-1-AP, Wuhan, China); anti-CD81 (SAB, 41779-2, College Park, USA); anti-CD63 (Abcam, ab68418, Cambridge, UK); anti-TSG101 (Abcam, ab83) and anti-β-actin (Proteintech, 20536-1-AP). PCR buffer for annealing, T7 RNA polymerase, T7 polymerase buffer, murine RNAse inhibitor, and nucleotide triphosphates (NTPs) used to obtain crRNA and NEBuffer 2.1 were purchased from New England Biolabs (Ipswich, USA). NaCl, MgCl_2_ and HEPES were obtained from Sigma-Aldrich (Guangzhou, China). Other chemical reagents were procured from Guangzhou Chemical Reagents Factory (Guangzhou, China). All reagents used in this study were of analytical grade and were used directly without any purification. All solutions were prepared using RNAase-free water.

### HCR-CRISPR detection

DNA sequences of the HCR system were designed according to the literature and modified to include an aptamer region 39. In preparation, H1 and H2 were heated separately to 95 °C for 2 min and then cooled to room temperature for 1 h. Then, H0, H1 and H2 were mixed together at concentrations of 0.5, 2 and 2 μM, respectively, in reaction buffer (100 mM Na_2_HPO_4_, 1 mM NaCl and 5 mM EDTA, pH 7.0) and incubated at 37 °C for 1 h. Agarose gels (2%) and PAGE gels were run to confirm the formation of HCR products. After Goldview (Maygene, Guangzhou, China) staining, the gels were transferred to the Tanon-2500 (Shanghai, China) for gel imaging.

For a standard CRISPR-Cas12a method, crRNAs were transcribed *in vitro* as previously reported 31, 55. The Cas12a-crRNA duplex was preassembled by mixing 250 nM Cas12a and 500 nM crRNA and performing target recognition in 1× NEBuffer 2.1. After incubation for 30 min at 37 °C, 50 nM collateral ssDNA was added, and the mixture was incubated in a fluorescent plate reader (Tecan spark, Shanghai, China) for 0.5-2 h at 37 °C, with the fluorescence intensity (FI) measured every 5 min (ssDNA reporter = λ_ex_: 535 nm; λ_em_: 572 nm).

For heterogeneous HCR-CRISPR, H0 at a specified concentration was added to the 0.72 mg/mL streptavidin MBs and incubated at 37 °C for 0.5 h followed by three washes. Then, the beads were resuspended with 50 μL of 2 μM H1 and 50 μL of 2 μM H2 and incubated for 1 h at 37 °C. After three washes, we carried out the standard CRISPR-Cas12a method.

### Cell culture, cell-derived EV isolation and EV characterization

The human nasopharyngeal carcinoma cancer (NPC) cell line SUNE2 (Chinese Academy of Sciences, Shanghai, China) was cultured in RPMI-1640 medium (Invitrogen, Carlsbad, USA) containing 10% heat-inactivated FBS (Thermo Fisher, Rockford, IL, USA) at 37 °C with 5% CO_2_. EVs secreted from SUNE2 cells were isolated by the ultracentrifugation method as described in our previous study 21. Briefly, cells were cultured to 70-80% confluence and subsequently washed three times with PBS. Then, the cells were maintained in media without FBS for 24 h. All culture media were collected for EV isolation by ultracentrifugation at 100,000 ×g for 75 min at 4 °C twice and resuspended in PBS.

The isolated EVs were observed using a JEM-1200EX transmission electron microscope (TEM) (JEOL, Japan), and the concentration and size were verified by nanoparticle tracking analysis (NTA) performed with a NanoSight NS300 (Malvern Instruments Ltd, UK). The characteristic proteins of the EVs were analyzed by Western blotting as previously reported 21.

### Apta-ELISA

Next, 100-µL serial dilutions of SUNE2-derived EVs (10 -10^6^ particles/µL) were added to the anti-CD63, anti-CD81 and anti-CD9 MBs and incubated with 100 μL of 500 nM biotin-labeled aptamer at 37 °C for 1 h. After washing three times, 100 μL of diluted streptavidin-HRP (1:200 in PBS, R&D Systems, Minnesota, USA) was added per well, followed by incubation for 20 min at room temperature. The MBs were washed three times with wash solution (0.01% Tween 20 and 5 mM MgCl_2_ in PBS) between each binding incubation. Finally, the 100-μL substrate (tetramethylbenzidine) to HRP was added, followed by incubation at 37 °C for 10-15 min in the dark. The reaction was stopped with 50 μL of 2 N H_2_SO_4_, and the OD was measured immediately at a wavelength of 450/630 nm.

### Apta-HCR-ELISA

Prior to each experiment, biotin-labeled H1 and H2 stock solutions were heated to 95 °C for 2 min and then allowed to cool to room temperature for 1 h before use. SUNE2-derived EVs were captured and recognized by 500 nM H0 using the same protocol for the apta-ELISA. At the same time, 50 μL of 2 μM H1-biotin and 50 μL of 2 μM H2-biotin were mixed with 5 μL of 10 μg/mL streptavidin-HRP and incubated for 0.5 h at room temperature. Next, EV-captured beads with the aptamer bound were washed three times with PBST followed by resuspension in the above mixture of HRP-labeled H1 and H2 and incubated for 1 h at 37 °C. The subsequent steps and OD measurement were the same as those for the apta-ELISA.

### Apta-HCR-CRISPR

First, 100-μL serial dilutions of the EVs (64-10^6^ particles/µL) were captured and recognized by 500 nM H0 using the same protocol as for the apta-ELISA. Then, the beads were resuspended with 50 μL of 2 μM H1 and 50 μL of 2 μM H2 and incubated for 1 h at 37 °C; after washing, the HCR products contained repetitive sequences bound to the beads, serving as targets for subsequent CRISPR-Cas12a recognition. Then, the standard CRISPR-Cas12a method was performed.

Clinical samples (50% dilution; 50 µL of serum in 50 µL of PBS) were also processed with a similar protocol to that described above.

### Clinical characteristics and ethics statement

For the NPC detection experiment, pretreated serum from 20 pathologically confirmed NPC patients was obtained from Sun Yat-sen University Cancer Center (SYSUCC), and 20 volunteers who underwent routine physical examinations with negative results were also selected from the health examination department at our center. To evaluate PD-L1^+^ EV changes after immunotherapy initiation, ten advanced cancer patients with serum collected within 7 days before (day -7) and after 4 courses of nivolumab or pembrolizumab therapy were enrolled and analyzed.

The serum from the enrolled subjects was centrifuged at 3600 rpm for 8 min and then stored at -80 °C until use. The clinical characteristics of the patients with NPC and the healthy controls are shown in Table S2, and the details of the patients treated with PD-1 therapy are shown in Table S3.

This study was reviewed and approved by the Institutional Review Board and Ethics Committee of SYSUCC (GZR2018-197). Written informed consent for the use of clinical parameters and collected samples for further studies at the time of patient admission was obtained as a general standard procedure; the samples were anonymous and de-identified before use.

### Statistical analysis

Statistical analyses were performed using GraphPad Prism version 8.0 software and Microsoft Excel. A two-tailed Student's t-test was used to analyze differences between the two groups. To evaluate the value of PD-L1^+^ EVs for immunotherapy surveillance, a paired t-test was applied. The diagnostic efficacy of nucleolin^+^ EVs was evaluated using the area under the receiver operating characteristic (ROC) curve (AUC). All tests were two tailed, and a *p*-value less than 0.05 was considered statistically significant. Three replicates were performed to improve the statistics.

## Results and Discussion

### Scheme

**Figure 1:**
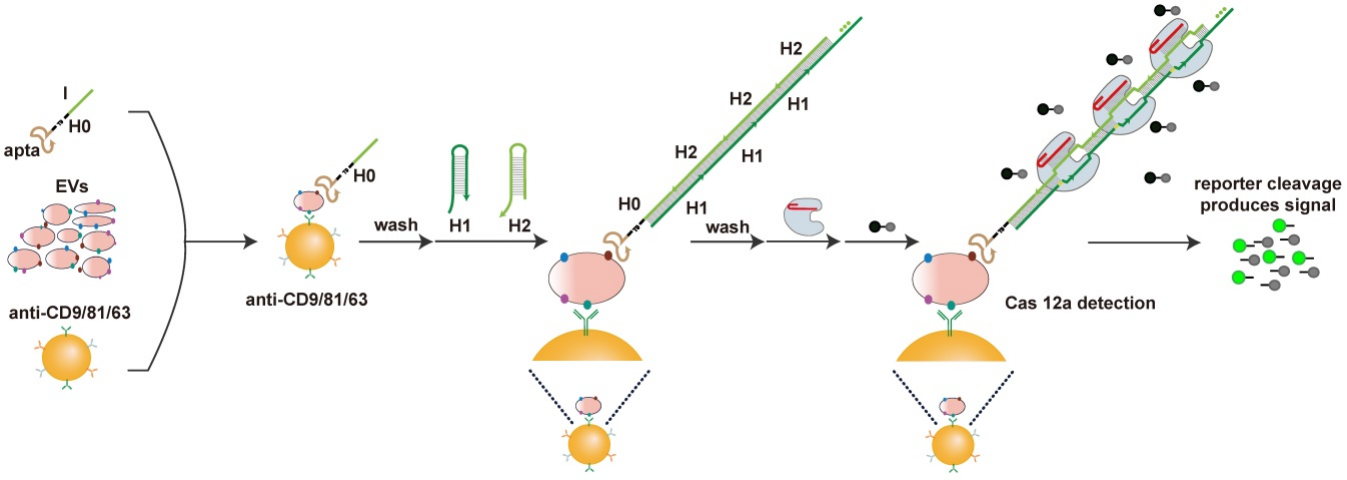
** Schematic of apta-HCR-CRISPR.** The EVs are captured by a cocktail of anti-CD63-, anti-CD81- and anti-CD9 antibody-coated beads and recognized with H0. The formed antibody-EV-H0 complexes trigger HCR and generate long repetitive target sequences that are specifically recognized by the added crRNA/Cas12a duplex. Target-activated Cas12a trans-cleaves nearby ssDNA-FQ reporter, resulting in readable and accumulating fluorescence signal proportional to the concentration of target positive EVs.

Our apta-HCR-CRISPR assay comprised three processes (Figure 1): aptamer-based ELISA (apta-ELISA) for EV capture and target recognition, HCR amplification and CRISPR-Cas12a DNA detection. In this proof-of-principle work, the initiator strand H0 was designed with three functional domains, including an aptamer for receptor recognition (apta), a nine-T spacer for structural steric hindrance diminishment (T) and an ssDNA region to initiate HCR (I), serving as the bridge for protein target recognition and DNA signal amplification. First, we captured EVs with a cocktail of anti-CD63-, anti-CD81- and anti-CD9 antibody-coated beads and recognized the targets with H0. If the EV membranes express target proteins, H0 binds to the target with the help of the corresponding aptamers and forms antibody-protein-H0 complexes, opening up H1s; this triggers a cascade reaction, which opens up H2s to form a long-nicked dsDNA with tens to hundreds of repetitive units containing protospacer adjacent motif (PAM) structures and target sequences for specific recognition by the predesigned crRNA. After washing off unbound nucleic acids, the added crRNA/Cas12a duplex binds the repetitive units in H1/H2 within the HCR assembly and induces the trans-cleavage activity of Cas12a, resulting in nearby nontarget ssDNA cleavage. Here, an ssDNA probe labeled with a donor fluorophore (FAM) at the 5' end and a quencher (BHQ) close to the 3' end was introduced as a reporter (FQ probe). When the reporter is intact, the proximity of the FAM to the BHQ suppresses its fluorescence, primarily via the Förster-type of energy transfer 56. Once target-activated Cas12a trans-cleaves the ssDNA reporter, a fluorescent signal will be produced due to the separation of the FAM from the BHQ, and the fluorescence will be proportional to the target concentration. In the absence of the target protein on EVs, H0 is washed away because no target is available to bind, resulting in no target DNA for Cas12a trans-cleavage and thus no change in the fluorescence signal. As a result, the concentration of the EV target protein can be calculated by monitoring the fluorescence change in the system. Using this strategy, we can transform the CRISPR-Cas12a system into a general sensor for quantification of a wide range of EV proteins.

### Feasibility of the HCR-CRISPR

**Figure 2:**
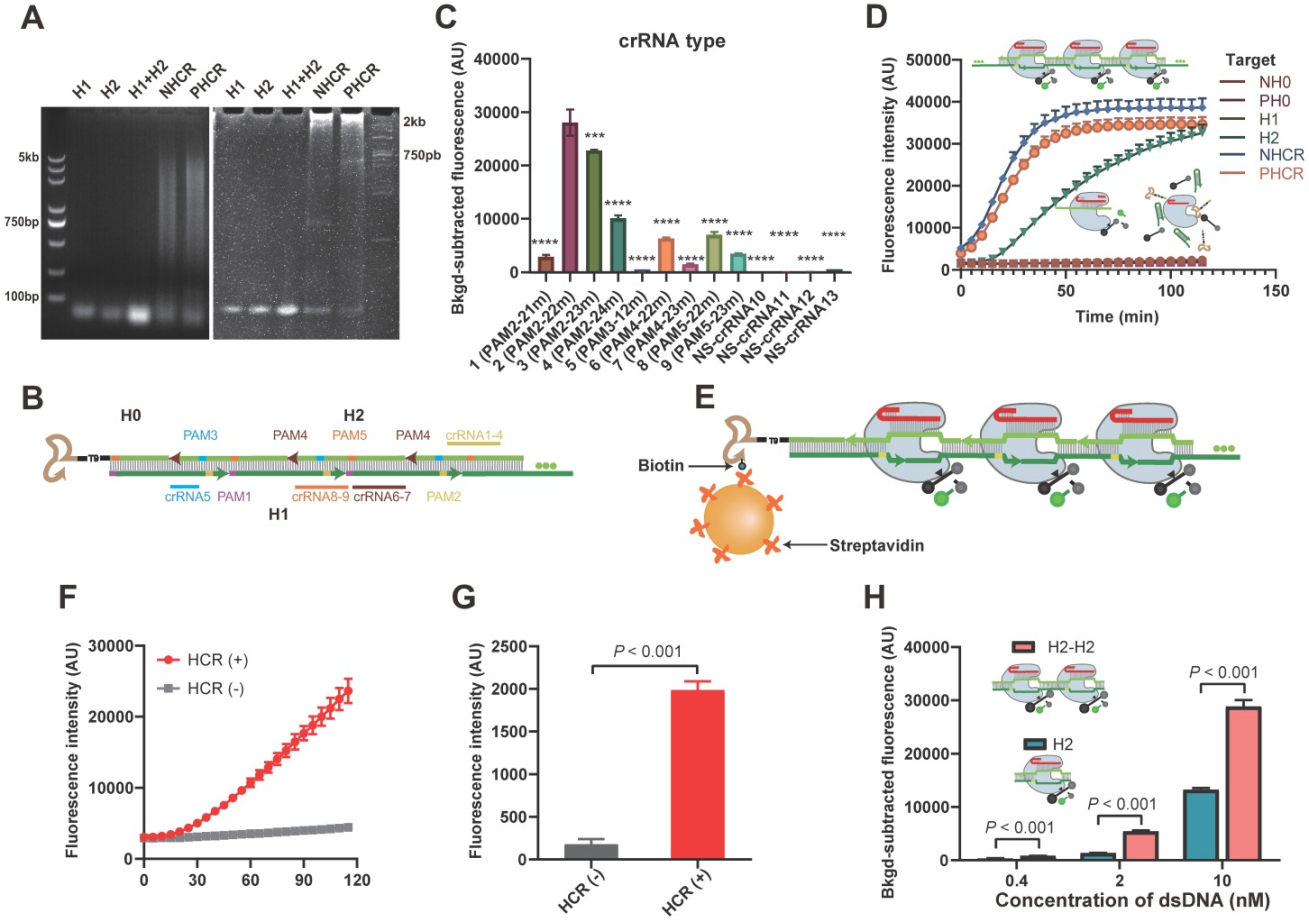
** Establishment of the HCR-CRISPR assay. (A)** Agarose gel (left) and PAGE (right) images of the HCR nucleic acid amplification assay. Lane 1, 2.0 μM H1; lane 2, 2.0 μM H2; lane 3, 2.0 μM H1 and 2 μM H2 mixture; lane 4, NHCR, 0.5 μM nucleolin H0 mixed with a mixture of 2 μM H1 and 2 μM H2; lane 5, PHCR, 0.5 μM PD-L1 H0 mixed with a mixture of 2 μM H1 and 2 μM H2. **(B)** Diagram of the H1/H2 sequences within the HCR targeted by Cas12a and the respective crRNA. Highlighted bases indicate the 5′ PAM sequence. The same color represents a paired crRNA and PAM. **(C)** Observed FI of HCR-CRISPR using different crRNAs to target 1μL of nucleolin HCR (NHCR) products. The nonspecific (NS) crRNA controls showed a low or zero value after subtracting the background FI. The *P* values were calculated by comparison with the crRNA2 group using a one-way ANOVA followed by a Sidak multiple-comparisons test. *** and **** represent *P* < 0.001 and *P* < 0.0001, respectively. **(D)** Representative real-time fluorescence kinetic measurement of CRISPR-Cas12a using different targets: nucleolin H0 (NH0), PD-L1 H0 (PH0), H1, H2, NHCR and PD-L1 HCR (PHCR) products. Fluorescence measurements were taken every 5 min for 2 h. Error bars represent the mean ± SD, where n = 3. **(E)** Heterogeneous HCR-CRISPR mechanism. Real-time fluorescence kinetic measurement **(F)** and the observed FI **(G)** of the heterogeneous HCR-CRISPR. HCR (+): 50 nM H0 mixed with a mixture of 2 μM H1 and 2 μM H2; HCR (-): 0 nM H0 mixed with a mixture of 2 μM H1 and 2 μM H2. Unbound DNA sequences were discarded by washing three times. Error bars represent the mean ± SD, where n = 3. **(H)** Observed FI of CRISPR-Cas12a using 0.4 nM, 2 nM and 10 nM H2 dsDNA or duplicate consecutive H2 (H2-H2) as activators. dsDNA, double-stranded DNA. Error bars represent the mean ± SD, where n = 3. FI, fluorescence intensity. For G and H, statistical analyses were performed using a two-tailed Student's t-test.

Prior to conducting the apta-HCR-CRISPR, we set up HCR-CRISPR using nucleolin and PD-L1 as examples. The feasibility of HCR was validated by both agarose and polyacrylamide gel electrophoresis (PAGE) (Figure 2A). As Cas12a endonuclease from *Francisella novicida* (Fn) exhibits a fast cleavage effect on dsDNA 57, 58, we expressed and purified FnCas12a (Figure S1) and developed our platform based on it.

The composition and length of complementarity between the crRNA and DNA largely modulate Cas12a nuclease activation 40, 59. Hence, prudent crRNA design and repeated testing are required to optimize the cleavage effect. To maximally trigger Cas12a endonuclease activity, crRNA must be adjacent to the PAM sequence of the target in its 3' region. As shown in Figure 2B, five PAMs (highlighted region) were found in the HCR dsDNA assembly. Considering that introducing a nick near the 5' region of the PAM could perturb DNA rigidity 60 and affect Cas12a-induced DNA bending for cleavage to slow down cleavage, PAM1 was skipped for the crRNA design. For the remaining four PAMs, we designed nine crRNAs covering different compositions and lengths, as shown in Table S1 and Figure 2B. Similar to previous reports, 22m and 23m crRNA-DNA heteroduplexes achieved good cleavage rates, especially PAM2-22m and PAM2-23m 29, 59. Cas12a/crRNA cleavage events also occurred in the 21m and 24m crRNA-DNA duplex group. However, 12 matched bases (12m) between crRNA5 and the target showed almost no Cas12a/crRNA cleavage events (Figure 2C), which is consistent with previous studies reporting that fewer than 15 base pairs of the crRNA-DNA heteroduplex was not sufficient to trigger NTS cleavage of Cas12a 29, 59. Nonspecific crRNAs (NS-crRNA10-13) were used as negative controls, and no fluorescence changes were observed. The crRNA2-H2 assembly at the PAM2 proximal end triggered the best catalytic activity and was selected as the universal DNA barcode for the next assay (Figure 2C). The PAGE result in Figure S2 confirmed that the Cas12a-crRNA2 duplex largely cis-cleaved the HCR products.

Given that HCR produces nicked dsDNA structures, to explore whether the nicks affect Cas12a-crRNA2 duplex cleavage events, we introduced ligase to close the nicks (Figure S3A). A standard CRISPR-Cas12a assay was performed with both sets (with and without nicks) of nucleolin HCR products (NHCR) at 10-fold and 50-fold dilutions, and no significant differences were found between the two sets (Figure S3B), indicating that nicks on the HCR products did not prevent cleavage events. Based on these data, our HCR-CRISPR assay was successfully established and worked well without preligation.

Next, target specificity was explored using H1, H2, and H0. We observed that the fluorescence intensity (FI) increased with the reaction time for Cas12a-crRNA cleavage in both the HCR products and the H2 ssDNA group but not in the H1 ssDNA and H0 groups (Figure 2D), which is consistent with previous reports showing that both crRNA-complementary ssDNA (here, H2) and dsDNA (here, HCR products) could activate Cas12a to cleave the ssDNA-FQ reporter substrate 29. We further designed our platform in a heterogeneous assay format using solid beads and included washing steps to remove unbound H2. As illustrated in Figure 2E, streptavidin-MBs captured biotinylated H0, triggering HCR amplification to form long repetitive dsDNA structures. After excess H1 and H2 were removed by washing, the bound products were detected by CRISPR-Cas12a. The fluorescence kinetic curves showed that the FI increased in the HCR group but was barely evident in the control group (Figure 2F). Moreover, the FI value in the HCR group was substantially stronger than that in the negative control group (Figure 2G), indicating that HCR and CRISPR-Cas12a can work successfully in a solid-phase format.

Considering that our methodology is based on the speculation that a repeated sequence comprising multiple crRNA targetable barcodes confers Cas12a with higher collateral cleavage activity than a single target sequence, we chemically synthesized duplicate consecutive H2 dsDNA as activators (H2-H2) and considered H2 to be the cognate target. The fluorescence signals in duplicate consecutive H2 dsDNA were almost twice those in individual H2 dsDNA at three concentrations (Figure 2H), suggesting that the sequentially repetitive target sequences amplify the CRISPR/Cas12a signal. Similar results were also observed for ssDNA (Figure S4). We also confirmed that the gap was long enough to not hinder Cas12a-crRNA target recognition events (Figure S5 and supplementary information). These results laid a foundation for our HCR-based CRISPR amplification system.

Cas12a cleavage could be improved by buffer, crRNA, Cas12a concentration, and reaction temperature optimization (Figure S6 and supplementary information). With these optimized experimental conditions, we could detect a synthetic target dsDNA and target ssDNA with limits of detection (LODs) of 0.01 nM and 0.5 nM (Figure S7), respectively, which are similar to those in previous CRISPR/Cas12a-Dx studies, and the LOD was lower when a dsDNA activator was bound because of the higher Cas12a trans cleavage efficiency 29. Our HCR-CRISPR platform also used dsDNA activators and was therefore likely to be more sensitive than the technique using an ssDNA target assembly.

### Comparison of apta-ELISA, apta-HCR-ELISA and apta-HCR-CRISPR

Prior to the detection of EV-derived proteins using the proposed apta-HCR-CRISPR platform, nasopharyngeal carcinoma cancer (NPC) EVs secreted from SUNE2 cells were harvested. The enriched EVs were confirmed to be cup-shaped vesicles with diameters of approximately 100 nm and were positive for the CD63, CD9, TSG101 and CD81 markers (Figure S8), indicating the successful extraction of NPC EVs.

**Figure 3:**
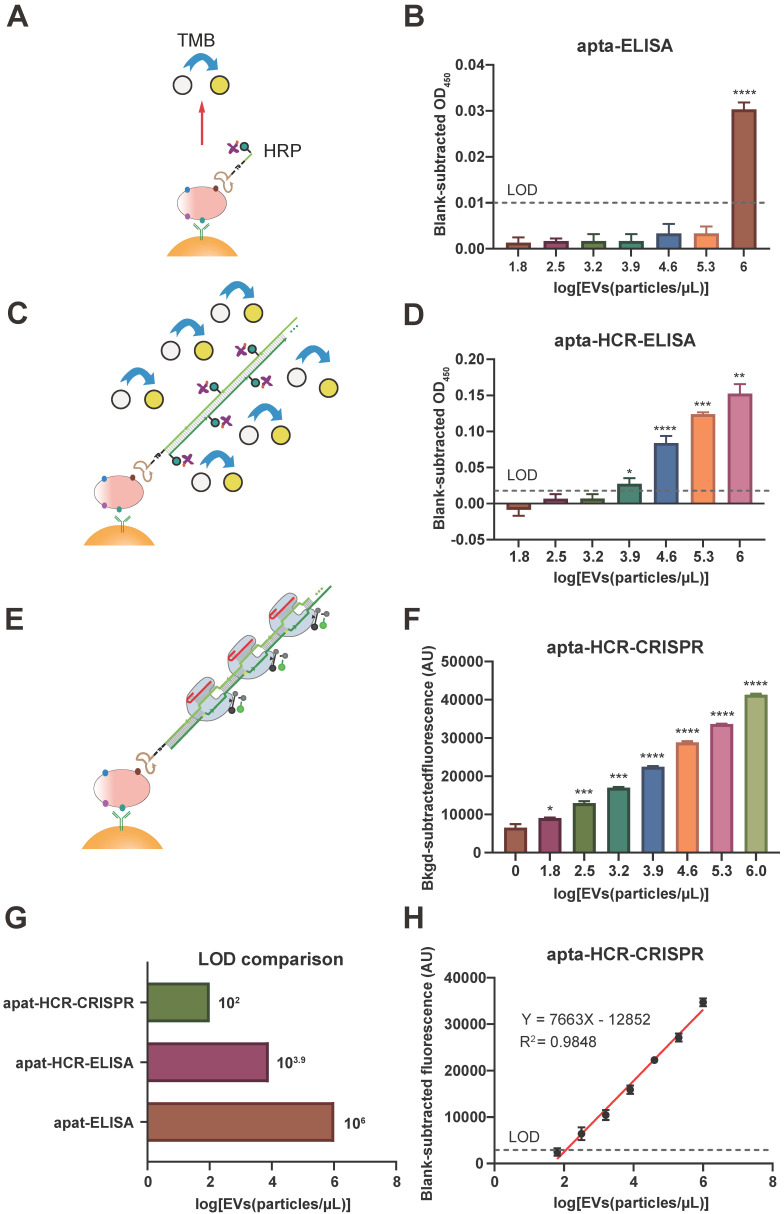
** Comparison of the apta-ELISA, apta-HCR-ELISA and apta-HCR-CRISPR assays. (A)** apta-ELISA mechanism. In the apta-ELISA assay, EVs were added to the anti-CD63, anti-CD81 and anti-CD9 MBs, incubated with a biotinylated aptamer, and washed and resuspended in streptavidin-HRP. The reaction was launched by adding the substrate, and the OD was proportional to the original concentration of target positive EVs. **(B)** Detection of nucleolin^+^ EVs by apta-ELISA with serial concentrations of SUNE2 EVs spiked in PBS from 64-10^6^ particles/µL. **(C)** apta-ELISA-HCR mechanism. EVs were added to the anti-CD63, anti-CD81 and anti-CD9 MBs, incubated with a biotinylated aptamer, and washed and resuspended in premixture HRP-labeled H1 and H2. The reaction was launched by adding the substrate, and the OD was proportional to the original concentration of target positive EVs. **(D)** Detection of nucleolin^+^ EVs by apta-HCR-ELISA with serial concentrations of SUNE2 EVs spiked in PBS from 64-10^6^ particles/µL. **(E)** apta-HCR-CRISPR mechanism. Based on the apta-ELISA-HCR assay, the HCR products were targeted by Cas12a/crRNA duplex and triggered Cas12a to cleave the ssDNA-FQ reporter substrate, resulting in readable and accumulating FI proportional to the concentration of target positive EVs. **(F)** Detection of nucleolin^+^ EVs by apta-HCR- CRISPR with serial concentrations of SUNE2 EVs spiked in PBS from 64-10^6^ particles/µL. **(G)** Comparison of the LOD of apta-HCR-CRISPR, apta-HCR-ELISA and apta-ELISA in detecting nucleolin^+^ EV spiked in PBS. **(H)** The concentration change in nucleolin^+^ EVs is linearly related to the FI through fitting curves, Y= 7663 lg (EVs) - 12852 (R^2^ = 0.9848). FI, fluorescence intensity. PBS served as a blank. The *P* values were calculated using a one-way ANOVA followed by a Sidak multiple-comparison with the former group. *, **, *** and **** represent *P* < 0.05,* P* < 0.01, *P* < 0.001 and *P* < 0.0001, respectively. Error bars represent the mean ± SD, where n = 3.

Our platform used aptamers as substitutes for antibodies to recognize surface proteins on EVs. Thus, we tested the feasibility of using the aptamers to recognize target proteins using apta-ELISA, and the mechanism is shown in Figure 3A. SUNE2 TEVs (64 - 10^6^ particles/µL) spiked in PBS were used as samples, and PBS served as a blank. After subtracting the PBS background OD, we found that the OD values of apta-ELISA were barely detectable until the EV concentration reached 10^6^ particles/µL for both nucleolin^+^ and PD-L1^+^ TEVs (Figure 3B, Figure S9A), indicating that the aptamers could recognize the corresponding proteins on TEVs but exhibited low sensitivity. To increase the sensitivity, we constructed an apta-HCR-ELISA to combine nucleic acid-based amplification and recognition events (the mechanism is shown in Figure 3C) and found that the LOD was enhanced 100-fold to approximately 10^4^ particles/µL (Figure 3D, Figure S9B). Despite the significant improvement in sensitivity, given that the average number of EVs in bodily fluids ranges from 1 × 10^2^ to 3 × 10^6^ particles/µL 61, further effort should be focused on improving the sensitivity for EV detection.

Therefore, we detected EV-derived proteins by combining the apta-ELISA and the highly efficient signal amplification HCR-CRISPR/Cas12a platform (Figure 3E). SUNE2 TEVs (10^6^ particles/µL) with 5-fold serial dilutions spiked in PBS (64 - 10^6^ particles/µL) were used as samples (Figure 3F), and an FI alteration was observed that gradually increased with increasing concentrations of EVs, with its alteration between adjacent groups being statistically significant (*P* < 0.05). The LOD of apta-HCR-CRISPR for nucleolin^+^ TEVs was 10^2^ particles/µL based on 3σ_b_ (σ_b_ is the standard deviation of the PBS, n = 3), which was approximately four orders of magnitude lower than that of the apta-ELISA and 100-fold lower than that of the apta-HCR-ELISA (Figure 3G, Figure S9C). A linear correlation between the concentration of nucleolin^+^ EVs (X) and the FI (Y) ranging from 64 to 10^6^ particles/µL was observed (Figure 3H), with the following equation: Y = 7,663 lg(X) - 12,852, R^2^ = 0.9848. Evidently, the increased sensitivity is due to both amplification by HCR and the turnover trans-cleavage effects of CRISPR-Cas12a. Similarly, our platform for PD-L1^+^ EV detection achieved a linear range of 64-10^6^ particles/µL and an LOD of 10^2^ particles/µL (Figure S9D, S9E). The linear equation was Y = 7895 lg(X) - 14376 (Figure S8E, R^2^ = 0.9652).

We believe that our approach is potentially suitable for analyzing TEVs in clinical applications given the low cost, easy operation, high sensitivity and wide linear range. A comparison of our proposed platform and recently reported methods for EV-derived protein detection is shown in Table S4. In general, our strategy is sensitive, convenient, and cost effective and requires no sophisticated equipment. Despite the capacities of our strategy, limitations are still present compared to the enzyme-assisted nucleic acid amplification-based methods, such as RPA-based or LAMP CRISPR/Cas12a assays, especially in terms of the kinetic efficiency. By controlling the optimal conditions, such as hairpins with relatively large loops, preparation by heating to 95 °C for 2 min, and using a 37 °C amplification temperature and HCR reaction buffer containing sufficient Na^+^, with the help of CRISPR-Cas12a, the HCR time could be reduced to 1 h with a low LOD of 10^2^ particles/µL.

### Apta-HCR-CRISPR assay for clinical samples and serum nucleolin^+^ and PD-L1^+^ EVs in clinical applications

**Figure 4:**
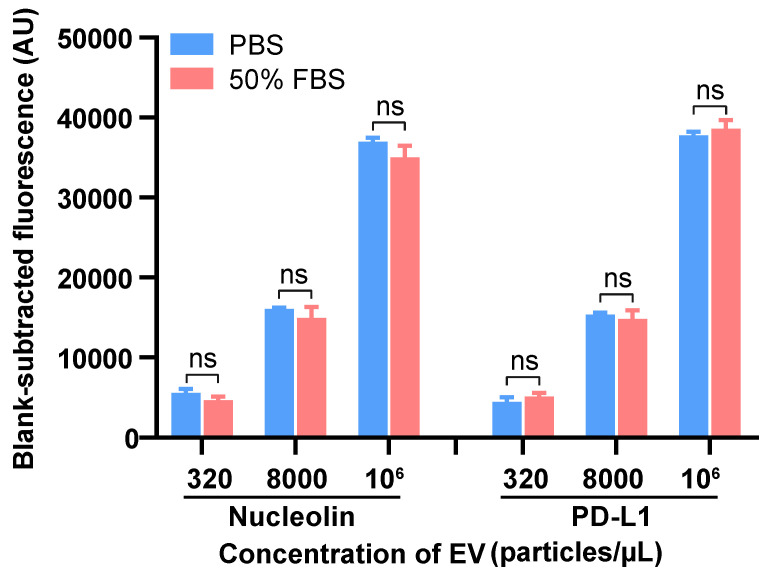
** Detection of the EVs in spiked EV-depleted FBS using the apta-HCR-CRISPR assay.** The FIs were blank subtracted. Statistical analyses were performed using a two-tailed Student's t-test. ns represents *P* > 0.05. Error bars represent the mean ± SD, where n = 3.

**Figure 5:**
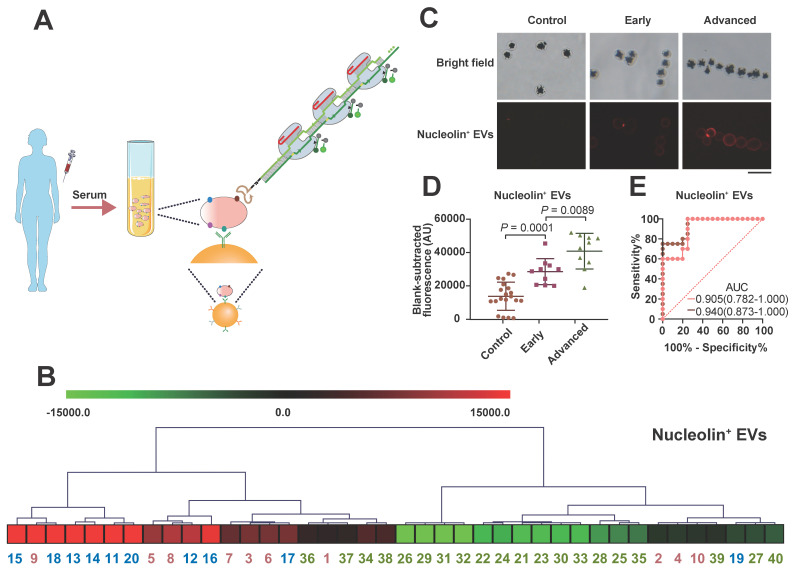
** Serum sample detection of nucleolin^+^ EVs with the apta-HCR-CRISPR assay. (A)** Mechanism of serum sample detection with the apta-HCR-CRISPR assay. **(B)** Non-supervised hierarchical clustering analysis of nucleolin^+^ EVs in early-stage and advanced-stage NPC patients and controls. Samples 1-10 were early-stage NPC, samples 11-20 were advanced-stage NPC, and samples 21-40 were the controls. **(C)** Representative immunofluorescence staining image of nucleolin proteins on serum EV membranes. Scale bar represents 100 µm. **(D)** Scatter plots of the nucleolin^+^ EV levels in the serum samples from the controls, early-stage NPC patients and advanced-stage NPC patients measured by the apta-HCR-CRISPR. The FIs measured for individual subjects were adjusted by the background and the blank. A statistical comparison of two groups was performed by a two-tailed Student's t-test. **(E)** ROC curve analysis evaluating the diagnostic power of nucleolin^+^ EVs to differentiate early-stage NPC (pink line) or early + advanced stage NPC (orange line) from controls.

We next tested the applicability of our method in complex serum samples by spiking EVs into 50% EV-depleted fetal bovine serum (FBS) at three different concentrations, and comparable results between the signal of the EVs spiked in FBS and those in PBS were observed (Figure 4). This assay achieved good recovery rates within the range of 83.7-115.6% with relative standard deviations (RSDs) of 2.8-9.1% for detecting both nucleolin^+^ and PD-L1^+^ EVs (Table S5). The results suggested that this platform has good reproducibility and high specificity with an acceptable sample matrix effect. As the procedure for our method includes washing steps as in a conventional ELISA, we can easily understand that the apta-HCR-CRISPR platform possesses an excellent anti-interference property. We further evaluated the sensitivity performance of our platform for complex samples. Similarly, the LOD of apta-HCR-CRISPR with serum was approximately 10^2^ particles/µL (Figure S10), surpassing that of apta-HCR-ELISA (approximately 10^4^ particles/ul) and apta-ELISA (10^6^ particles/ul). Therefore, the proposed assay can work well for complex biological samples and has great potential for use in clinical serum samples (Figure 5A).

Nucleolin has been identified to be markedly upregulated in NPC tissues by iTRAQ and tissue microarray methods and has been confirmed to be expressed on the surfaces of malignant cell-derived EVs 62. However, the value of EV-derived nucleolin has not been explored in NPC. NPC, which is prevalent in China, is a deadly cancer because most cases are diagnosed at late stages 63. To investigate their clinical value for early NPC detection, the serum levels of nucleolin^+^ TEVs in 20 healthy people, 10 early-stage (stages I and II) NPC patients, and 10 advanced NPC patients were measured with the apta-HCR-CRISPR assay. No significant differences in sex or age were found between the three groups. As shown in Figure 5B, the expression levels of nucleolin^+^ EVs varied across individual participants. Both immunofluorescence (IF, Figure 5C) and apta-HCR-CRISPR (Figure 5D) experiments showed that the levels of nucleolin^+^ EVs in early NPC patients were significantly higher than those in healthy controls (Figure 5D, *P* = 0.0001), and the advanced NPC group exhibited higher levels compared to the early-stage NPC group (Figure 5D, *P* = 0.0089), suggesting that nucleolin^+^ TEVs may be potential biomarkers for NPC diagnosis. Receiver operating characteristic (ROC) curve analysis and the area under the curve (AUC) were used to assess the diagnostic effect. A superior diagnostic effect (Figure 5E, AUC = 0.940) in differentiating NPC patients from control subjects was observed. The excellent accuracy (AUC = 0.905) was maintained even when discriminating early NPC patients from controls, indicating the potential clinical application value of nucleolin^+^ TEVs for early NPC diagnosis. We will continue to verify the performance of nucleolin^+^ TEVs in NPC screening in samples from larger patient cohorts.

**Figure 6:**
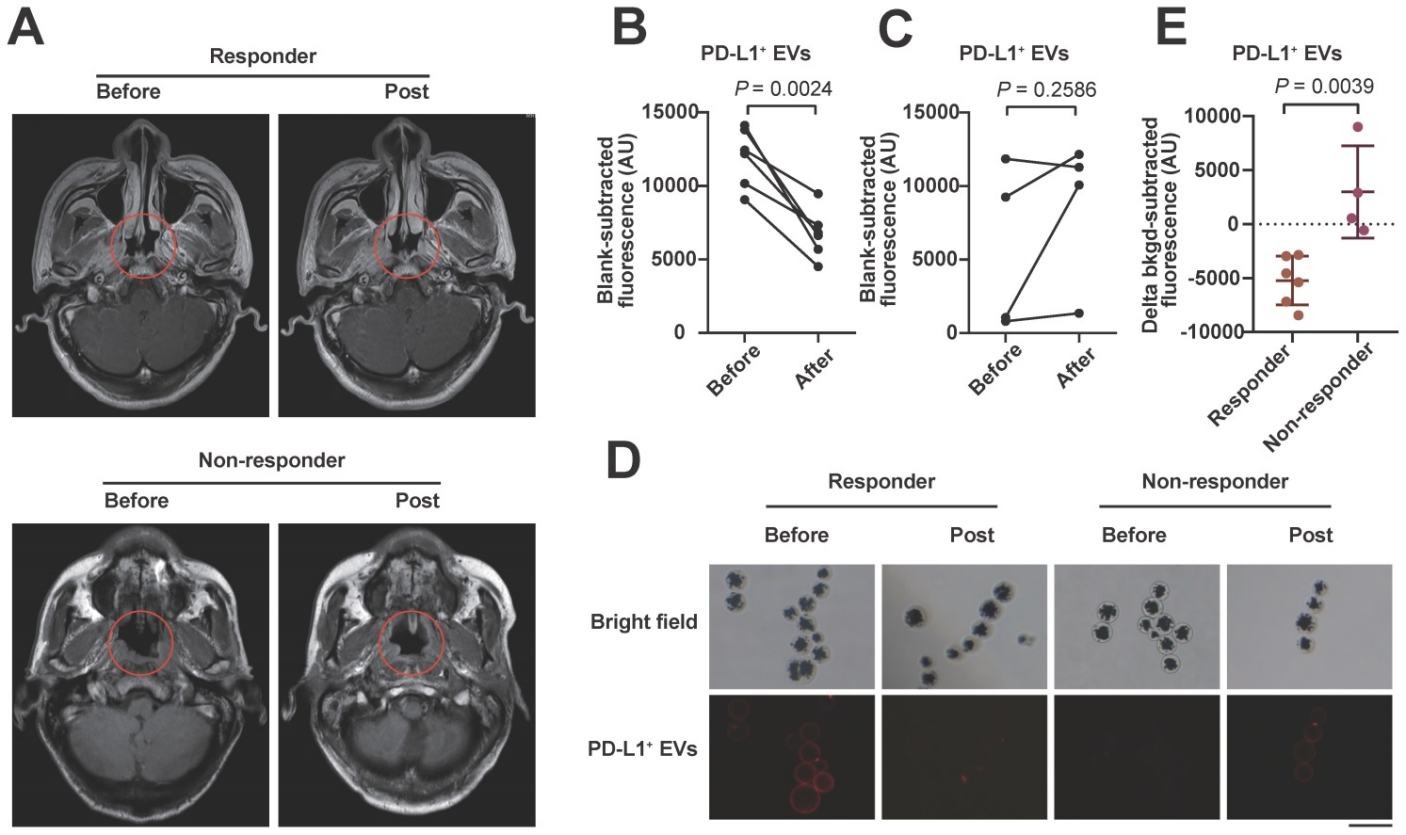
** Serum sample detection of PD-L1^+^ EVs with the apta-HCR-CRISPR assay. (A)** Representative MRI diagram of patients with different therapy responses. Upper panel shows the MRI images of a responder who had been assessed as having stable disease (SD): before treatment (left) and post-treatment (right). Lower panel shows the MRI images of a non-responder who had been assessed as having progressive disease (PD): before treatment (left) and post-treatment (right). Trend graph of PD-L1^+^ EV levels detected in the responders **(B)** and non-responders **(C)** before and after 4-course anti-PD-1 therapy. Statistical analyses were performed using a two-sided paired t-test. **(D)** Representative immunofluorescence staining image of PD-L1 proteins on serum EV membranes. Scale bar represents 100 µm. **(E)** Comparison of the changes in the levels of serum EV PD-L1 after 4 courses of anti-PD-1 therapy between responders (n = 6) and non-responders (n = 4). Statistical analyses were performed using a two-tailed Student's t-test. Abbreviations: FI, fluorescence intensity.

We also examined serum PD-L1^+^ EVs with the proposed assay in 10 stage IV tumor patients (including 6 responders and 4 non-responders; 8 NPC and 2 lung cancer patients) undergoing anti-PD-1 therapy. After 4 courses of anti-PD-1 treatment, the level of circulating EV PD-L1 decreased significantly in the clinical responders (Figure 6A, upper) but not in the non-responders (Figure 6A, lower) compared to the levels of EV PD-L1 at the pretreatment baseline (Figure 6B, 6C), which was also confirmed by the IF results (Figure 6D). The decline in EV PD-L1 levels stratified clinical responders from non-responders (Figure 6E,* P* = 0.0039). These results are consistent with those of other studies showing that dynamic changes in PD-L1 levels are ideal predictive biomarkers for immune checkpoint blockade therapy responses 9, 64, suggesting that our method may be useful for monitoring the efficacy of immunotherapy.

## Conclusions

In conclusion, we successfully constructed a novel apta-HCR-CRISPR assay to detect TEV surface proteins. Our method can detect TEV 10^2^ particles/µL directly from complex sample environments. The multiple HCR barcodes recognized by crRNA/Cas12a provide an easily designed and highly adaptable aptamer-based protein detection method. Considering its high sensitivity, cost efficiency and ease of operation, this structurally versatile and stable method has considerable potential to be exploited as a routine bioassay for efficient clinical diagnosis or therapy surveillance. Our experiment with a small sample preliminarily confirmed that serum nucleolin^+^ TEVs are potential biomarkers for NPC detection and that changes in PD-L1^+^ TEVs levels are potential predictive biomarkers for immune checkpoint blockade therapy responses.

## Supplementary Material

Supplementary figures and tables.

## Abbreviations

HCR: hybridization chain reaction
TEV: tumor-derived extracellular vesicle
crRNA: CRISPR RNA
PD-L1: programmed death ligand 1
LOD: limit of detection
NPC: nasopharyngeal carcinoma cancer
EVs: extracellular vesicles
PLA: proximity ligation assay
RPA: recombinase polymerase amplification
TMA: transcription-mediated amplification assay
CRISPR: clustered regularly interspaced short palindromic repeats
ssDNA: single-stranded DNA
PAGE: polyacrylamide gel electrophoresis
FBS: fetal bovine serum
FI: fluorescence intensity
NTA: nanoparticle tracking analysis
SYSUCC: Sun Yat-sen University Cancer Center
AUC: area under the curve
ROC: receiver operating characteristic
PAM: protospacer adjacent motif
Fn: Francisella novicida
NHCR: nucleolin HCR
apta-ELISA: aptamer-based ELISA
RSD: relative standard deviation
