# Supplementary material for: An ultrasensitive hybridization chain reaction-amplified CRISPR-Cas12a aptasensor for extracellular vesicle surface protein quantification
Source: Theranostics. 2020 Aug 13;10(22):10262–73. doi: 10.7150/thno.49047 (PMC7481432; doi:10.7150/thno.49047)
Supplement: Supplementary file 1 — Supplementary figures and tables. [file thnov10p10262s1.pdf]

Supplementary Information

An ultrasensitive hybridization chain reaction-amplified CRISPR-Cas12a aptasensor for extracellular vesicle surface protein quantification

Shan Xing[1,2#], Zedong Lu[1#], Qi Huang [1#], Huilan Li[1], Yu Wang[1], Yanzhen Lai[1,3], Yi He[1], Min Deng[4*], Wanli Liu[1*]

1 Department of Clinical Laboratory, State Key Laboratory of Oncology in South China, Collaborative Innovation Center for Cancer Medicine, Guangdong Key Laboratory of Nasopharyngeal Carcinoma Diagnosis and Therapy, Sun Yat-sen University Cancer Center, 651 Dongfeng Road East, Guangzhou 510060, P. R. China

2 School of Biomedical Engineering, Sun Yat-sen University, No. 132 Waihuandong Road, University Town, Guangzhou 510006, PR China

3 Heyuan People's Hospital, Heyuan, China

4 Affiliated Cancer Hospital & Institute of Guangzhou Medical University, No.78, Hengzhigang Road, Guangzhou 510095, P. R. China

[#] Shan Xing, Zedong Lu and Qi Huang contributed equally to this work

[*] **Corresponding authors:**

Wanli Liu: Department of Clinical Laboratory, Sun Yat-sen University Cancer Center, 651 Dongfeng Road East, Guangzhou 510060, Guangdong Province, China.

Telephone/Fax: +86 20 8734 3438

Email: liuwl@sysucc.org.cn

Min Deng: Affiliated Cancer Hospital & Institute of Guangzhou Medical University, No.78, Hengzhigang Road, Guangzhou 510095, P. R. China

E-mail: dengmin510095@163.com

Method section

Expression and purification of FnCas12a proteins

Escherichia coli Rosetta 2 (DE3) cells containing expression plasmids were used to express the FnCas12a protein according to a previous study [1]. IPTG was added to induce expression, and cells were incubated at 37 °C for 4 h. The harvested bacterial cells were centrifuged, and the precipitate was

obtained and then resuspended in lysis buffer (20 mM Tris-HCl, pH 7.5; 1 M NaCl; 20 mM imidazole; and 10% (v/v) glycerol), lysed by sonication and purified twice using an Abiotech nickel column and an Abiotech heparin column (Jinan, China). Finally, the FnCas12a protein was eluted with elution buffer containing 20 mM Tris-HCl (pH 7.5), 200 mM NaCl and 10% (v/v) glycerol. The protein was stored at -80 °C until use.

Effects on trans activity when nicks existed in the sequence

To investigate whether nicks in the sequence affect the trans activity, we used the DNA Ligation High Ver. 2 reagent (TOYOBO, Shanghai, China) to ligase HCR products. Briefly, DNA fragments were mixed with ½ volume of Ligation High Ver. 2 at 16 °C for 30 min. The probes and products were then stored at 4 °C for further use. Next, HCR products with or without nicks cleaved by the Cas12a-crRNA duplex over the indicated timepoints were analyzed, and the FI was also determined using the same protocol mentioned for HCR-CRISPR detection.

Western blotting analysis and immunofluorescence staining

The characteristic proteins of the EVs were analyzed by Western blotting as previously reported [2]. The dilution factors of the antibodies/aptamers were as follows: CD9 (1:1,000), CD81 (1:1,000), CD63 (1:1,000), nucleolin aptamer (500nM), and PD-L1 aptamer (500nM).

Immunofluorescence staining was also conducted as reported previously [2]. Briefly, the EVs were captured from serum by a cocktail of anti-CD9/CD63/CD81 MagBeads (Genscript, Nanjing, China) after incubation overnight at 4 °C. After washing three times, the beads were incubated with biotin-labeled nucleolin or PD-L1 aptamer (100 nM) for 1 h followed by incubation with streptavidin-Cy3 (1:1000, Invitrogen, Carlsbad, USA) for 30 min at room temperature. After 3 washes, the EVs were visualized using a fluorescence microscope (Olympus, TOKYO, Japan) by two independent observers.

Results and Discussion

The effect of gaps between adjacent target sequences in H2s on Cas12a-crRNA2 cleavage events

We chemically synthesized duplicate consecutive H2 (H2-H2) and H2-10 (5'-H2-10-H2-10-3') ssDNA as activators. The results showed that a gap of 10 reduced bases did not affect the activity of Cas12a-crRNA2 (Figure S5), indicating that our gap is suitable to avoid steric hindrance.

Optimization of experimental conditions for the HCR-CRISPR assay

We next optimized the experimental conditions for our assay to obtain an improved signal response. The selection of crRNA was described above. We investigated the chemical environment of the system for improved cleavage activity. An important factor that may affect Cas12a is the concentration of Mg^{2+}, which is thought to induce conformational coordination between the Cas12a RuvC domain and DNA by promoting the proximity of the Cas12a RuvC active cleavage site to the DNA [3]. Therefore, we investigated the effect of the Mg^{2+} concentration on cleavage activity. Cleavage activity was observed only in the presence of Mg^{2+} cations (Figure S6A). Increasing the concentration of Mg^{2+} ions up to 10 mM led to an enhanced fluorescence intensity (FI). Thus, an optimized Mg^{2+} concentration of 10 mM was used. Considering that Na cations allow the approximation of the phosphate groups of the DNA scaffold, which contribute to higher compaction of the DNA molecule [4-6] andmay prevent the Cas-crRNA target recognition-and-cleavage event, we also evaluated the effect of the Na^{+} ion concentration on the cleavage activity (Figure S6B). Concentrations of 0, 10, 50, 100, 200 and 300 mM were tested, and an interesting result that increasing the concentration of Na^{+} ions to greater than 50 mM prevented the cleavage activity was observed, which has not been reported before. The exact mechanism remains to be explored. Similar FIs were observed at 0, 10, and 50 mM Na^{+} concentrations. Given that a 50 mM Na^{+} concentration was

supported by previous studies [7, 8], we adopted this concentration for use in the following experiment. The pH condition affects the binding of enzymes and substrates by adjusting the dissociation state [9, 10], and Figure S6C shows the peak FI with pH 8.0 Tris-HCl buffer, which is similar to the previous studies [8, 11]. We also profiled the cleavage activity against the concentrations of crRNA and Cas12a. Using the control variable method, we observed that the concentration-dependent FI peaked at 500 nM crRNA and then stabilized (Figure S6D). Given that crRNA generation is relatively time consuming and expensive, we selected 500 nM crRNA for the present assay. Cas12a endonuclease at a concentration of 250 nM exhibited the most apparent and stable cleavage activity among the other groups (Figure S6E). Intriguingly, the cleavage activity decreased at a high level of Cas12a (500 nM), probably due to the steric hindrance caused by the large size of Cas12a [12]. We also attempted temperature optimization and found that the optimal temperature was 37 °C (Figure S6F), which was similar to the findings of previous studies [13].

The optimal conditions for HCR have been fully discussed, we adopted the following reaction conditions as reported by Dirks and Pierce [14]: 50 mM Na_2HPO_4/0.5 M NaCl (pH 6.8). These optimized experimental conditions were applied in the subsequent experiments.

Figures and Tables

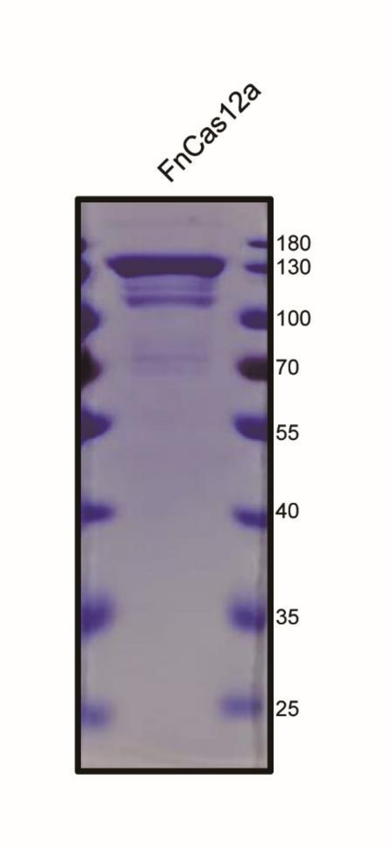

Figure S1. SDS-PAGE gel of purified FnCas12a.

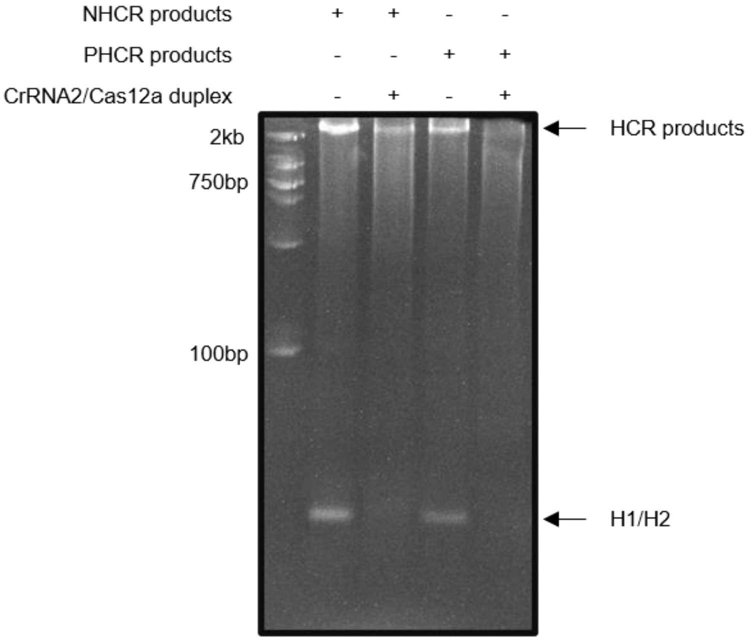

Figure S2. PAGE image of HCR products targeted by the Cas12a/CrRNA2 duplex. NHCR, nucleolin HCR; PHCR, PD-L1 HCR.

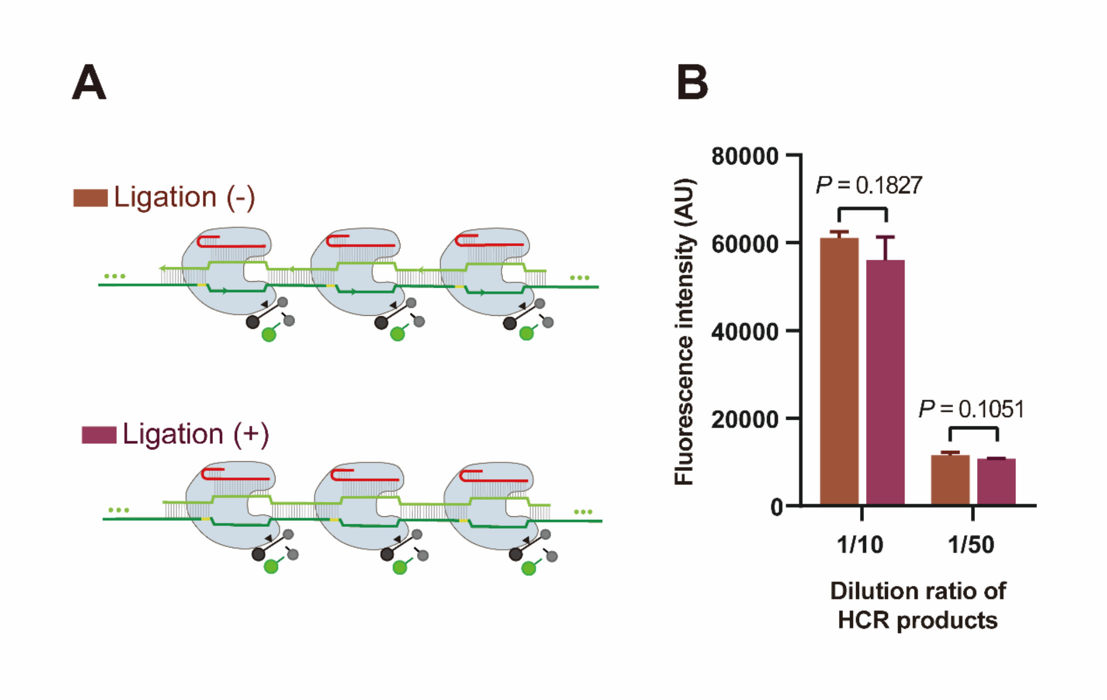

Figure S3. HCR products with or without ligation targeted by the Cas12a/CrRNA2 duplex.

(A) Schematic outlining the NHCR products ligated by a ligase to close the nicks.

(B) The observed fluorescence intensity of HCR-CRISPR/Cas12a using NHCR products at a 1/10 or 1/50 dilution as activators.

Statistical analyses were performed using a two-tailed Student's t-test.

Error bars represent the mean ± SD, where n = 3.

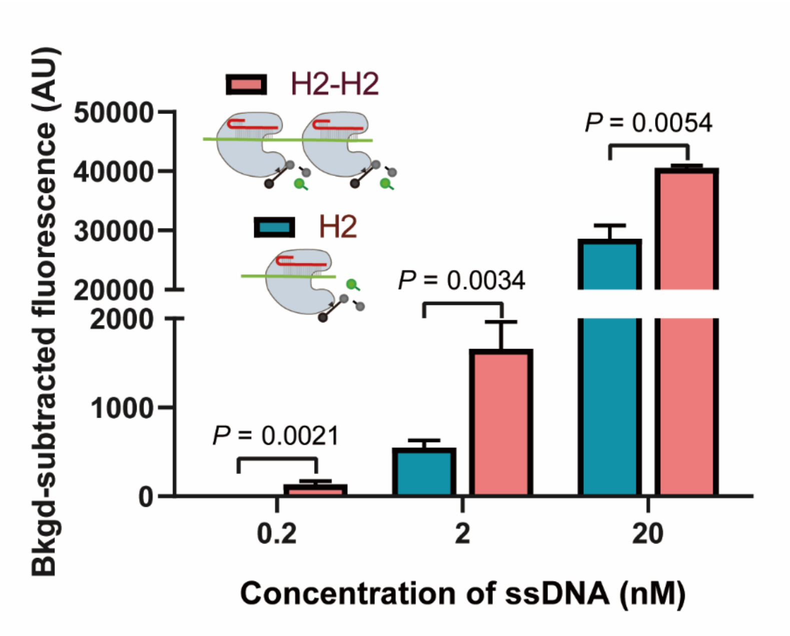

Figure S4. Repetitive ssDNA activators targeted by the Cas12a/CrRNA2 duplex. The observed fluorescence intensity of CRISPR-Cas12a using 1 nM, 5 nM and 20 nM H2 ssDNA or duplicate consecutive H2 as activators. ssDNA, single-stranded DNA. Statistical analyses were performed using a two-tailed Student's t-test. Error bars represent the mean ± SD, where n = 3.

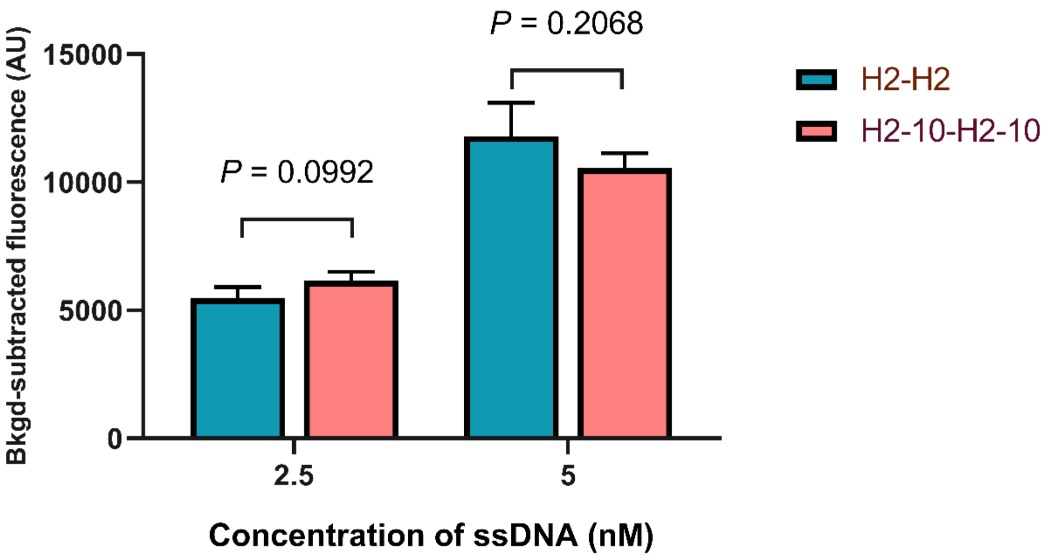

Figure S5. The effect of gap between adjacent target sequences in H2s on Cas12a-crRNA2 cleavage events. Statistical analyses were performed using a two-tailed Student's t-test. Error bars represent the mean ± SD, where n = 3.

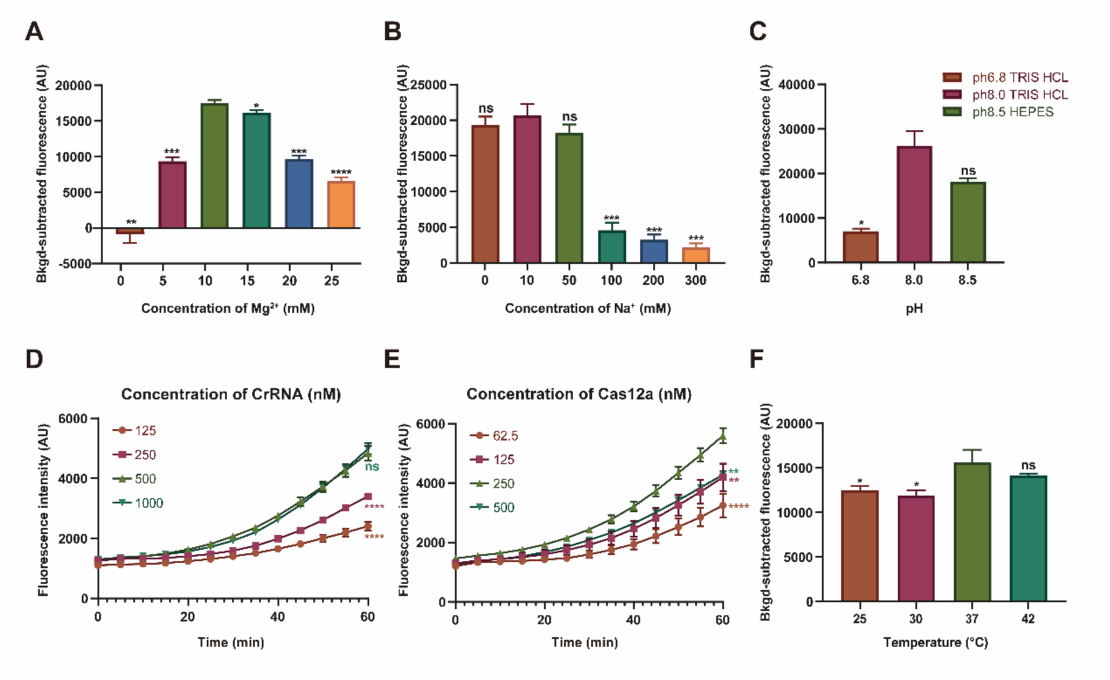

Figure S6. Optimization of the HCR-CRISPR assay.

Evaluation of the effect on FnCas12a collateral activity after targeting 40 nM H2 in a variety of buffers with different concentrations of Mg^{2+} (A) and Na^{+}(B) as well as different pH values (C). (D) Representative real-time fluorescence kinetic measurement of FnCas12a collateral activity after targeting 1 µL of 1/10 HCR products of different crRNA2 concentrations (250 nM Cas12a; 50 nM ssDNA reporter) and (E) different Cas12a concentrations (500 nM crRNA2; 50 nM ssDNA reporter).

(F) Temperature-dependent FI using FnCas12a.

P values were calculated using one-way ANOVA followed by a Sidak multiple-comparisons test with the optimal group. ns, *, **, *** and **** represent $P > 0.05$, $P < 0.05$, $P < 0.01$, $P < 0.001$ and $P < 0.0001$, respectively.

Error bars represent the mean ± SD, where n = 3.

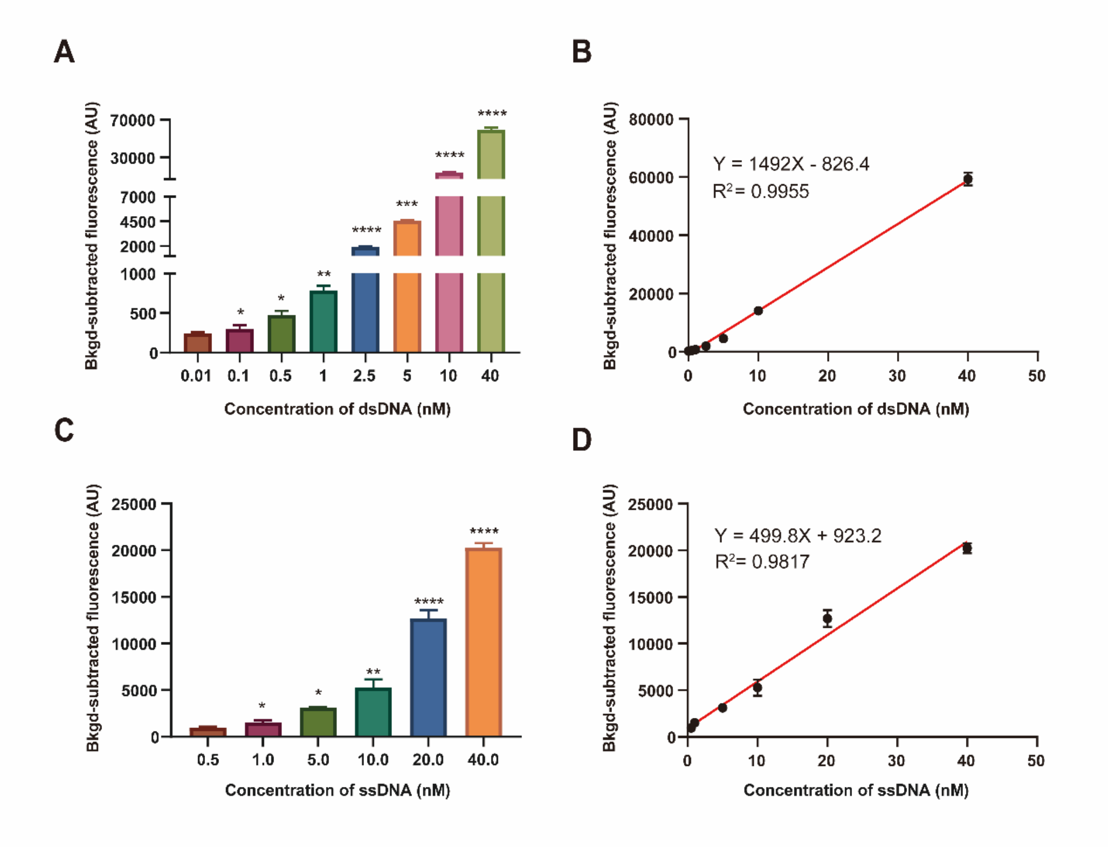

Figure S7. Sensitivity of the optimized CRISPR-Cas12a assay in detecting dsDNA and ssDNA. (A) Serial dilutions of the dsDNA template (0.01 - 40 nM) detected by the CRISPR-Cas12a assay. Assay time, 30 min. dsDNA, double-stranded DNA. (B) The concentration change in the dsDNA template is linearly related to the fluorescence intensity through fitting the following curve: Y = 1492X - 826.4 (R^2 = 0.9955). (C) Serial dilutions of the ssDNA template (0.5 - 40 nM) detected by the CRISPR-Cas12a assay. Assay time, 30 min. ssDNA, single-stranded DNA. (D) The concentration change in the ssDNA template is linearly related to the FI through fitting the following curve: Y = 499.8X + 923.2 (R^2 = 0.9817).

P values were calculated using one-way ANOVA followed by a Sidak multiple-comparisons test with the former group. ns, *, **, *** and **** represent $P > 0.05$, $P < 0.05$, $P < 0.01$, $P < 0.001$ and $P < 0.0001$, respectively.

Error bars represent the mean ± SD, where n = 3.

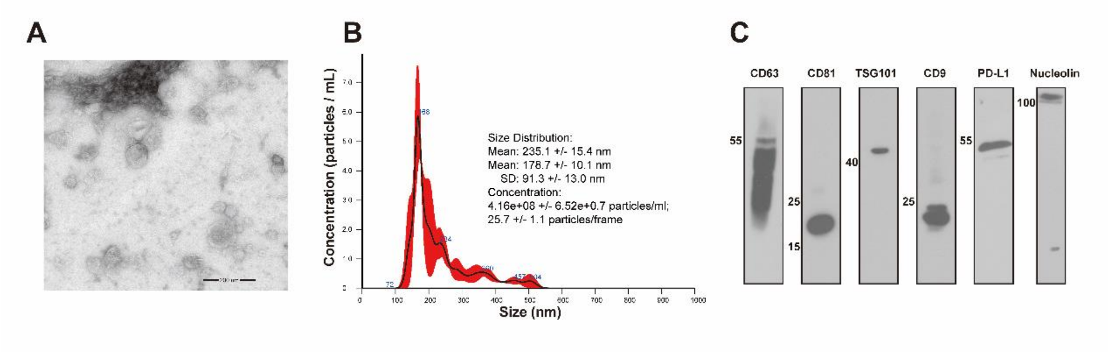

Figure S8. EV characteristics. (A) Representative TEM image of the isolated EVs from SUNE2 cells (scale bar: 200 nm). (B) NTA of isolated EVs with a peak of 168 nm and a calculated mean of 230.3 nm. (C) Western blotting demonstrating the presence of the proteins CD9, CD63, TSG101, CD81, nucleolin and PD-L1 in the EVs.

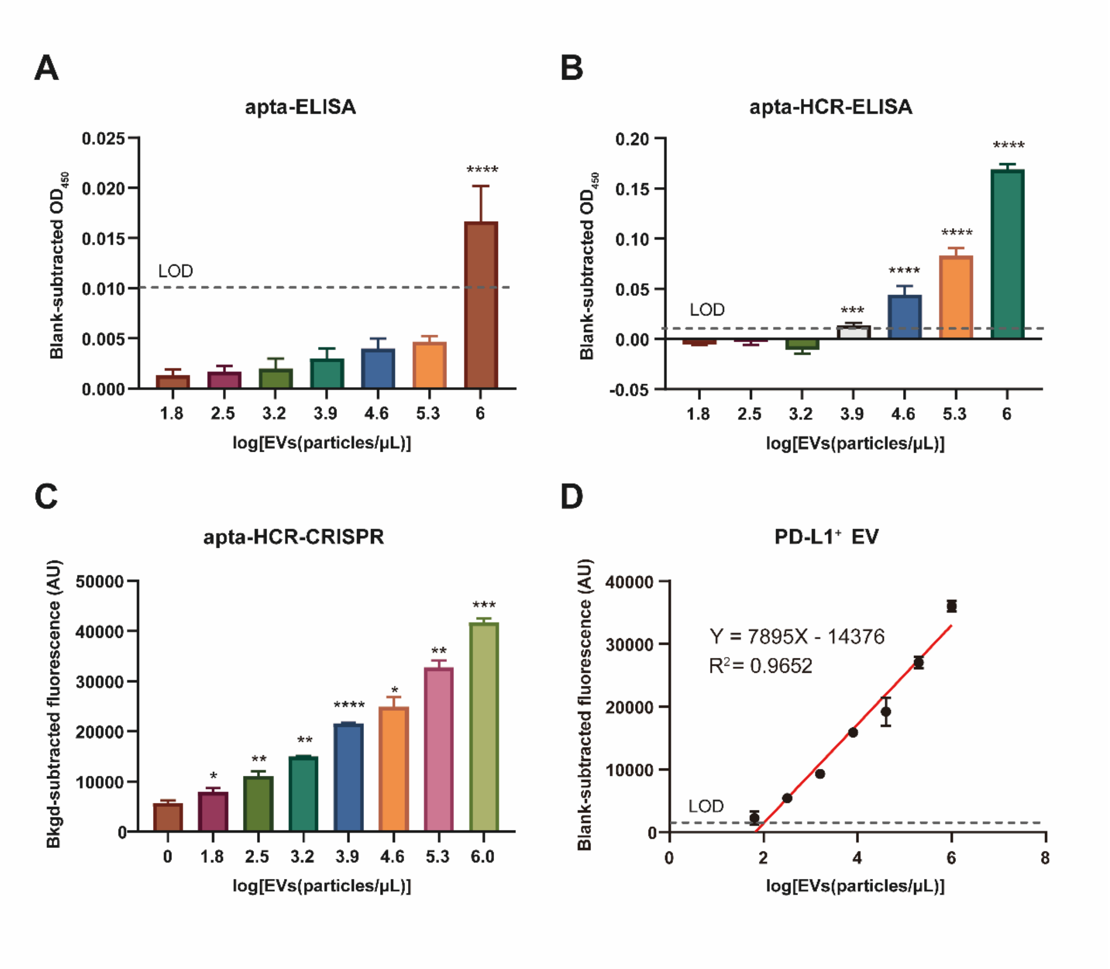

Figure S9. Comparison of the apta-ELISA, apta-HCR-ELISA and apta-HCR-CRISPR assays in detecting PD-L1$^+$ EVs spiked in PBS. (A) Detection of PD-L1$^+$ EVs by apta-ELISA with serial concentrations of SUNE2 EVs spiked in PBS between 64-10^6 particles/μL. (B) Detection of PD-L1$^+$ EVs by apta-HCR-ELISA with serial concentrations of SUNE2 EVs spiked in PBS between 64-10^6 particles/μL. (C) Detection of PD-L1$^+$ EVs by apta-HCR-CRISPR with serial concentrations of SUNE2 EVs spiked in PBS between 64-10^6 particles/μL. (D) The concentration change of PD-L1$^+$ EVs is linearly related to the FI through fitting the following curve: Y = 7895X - 14376 (R^2 = 0.9652).

PBS served as a blank. *P* values were calculated using one-way ANOVA followed by a Sidak multiple-comparisons test with the former group. *, **, *** and **** represent $P < 0.05$, $P < 0.01$, $P < 0.001$ and $P < 0.0001$, respectively.

Error bars represent the mean ± SD, where n = 3.

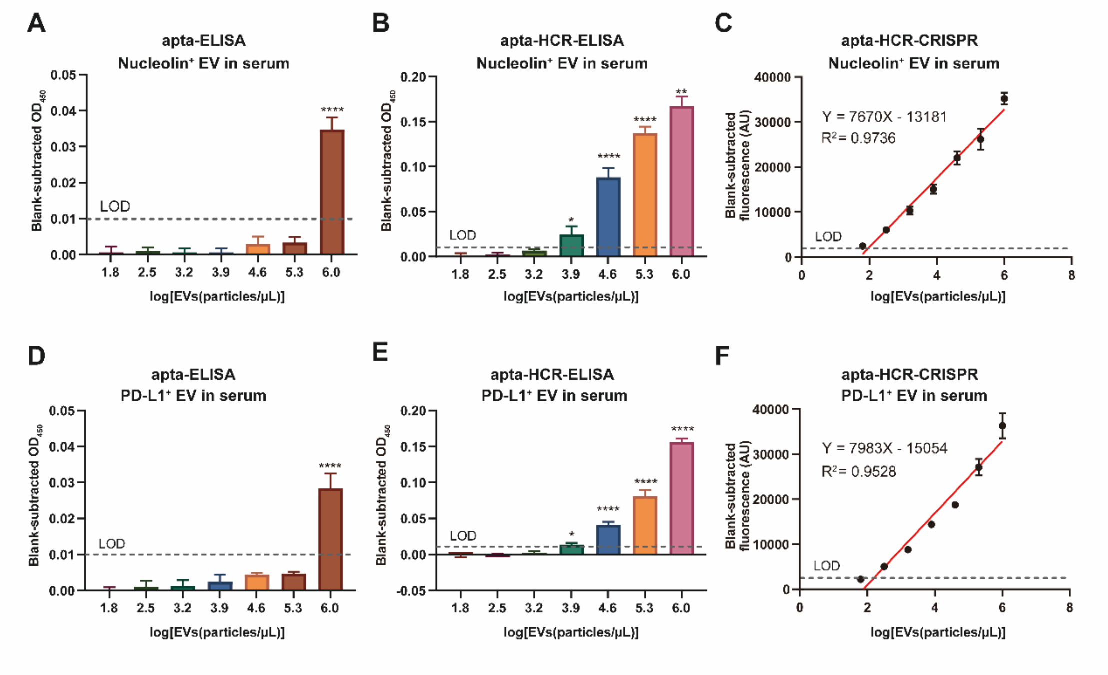

Figure S10. Comparison of the apta-ELISA, apta-HCR-ELISA and apta-HCR-CRISPR assay in detecting nucleolin⁺ and PD-L1⁺ EVs spiked in serum. (A) Detection of nucleolin⁺ EVs by apta-ELISA with serial concentrations of SUNE2 EVs spiked in 2× diluted serum from 64-10^6 particles/μL. (B) Detection of nucleolin⁺ EVs by apta-HCR-ELISA with serial concentrations of SUNE2 EVs spiked in 2× diluted serum from 64-10^6 particles/μL. (C) Correlation of the apta-HCR-CRISPR FI with the log EV concentration in detecting nucleolin⁺ EV spiked in a 2× diluted serum. (D) Detection of PD-L1⁺ EVs by apta-ELISA with serial concentrations of SUNE2 EVs spiked in 2× diluted serum from 64-10^6 particles/μL. (E) Detection of PD-L1⁺ EVs by apta-HCR-ELISA with serial concentrations of SUNE2 EVs spiked in 2× diluted serum from 64-10^6 particles/μL. (F) Correlation of the apta-HCR-CRISPR FI with the log EV concentration in detecting PD-L1⁺ EV spiked in a 2× diluted serum.

2× serum was derived from healthy participants without detectable target protein expression and served as a blank. *P* values were calculated using

one-way ANOVA followed by a Sidak multiple-comparisons test with the former group. *, **, *** and **** represent $P < 0.05$, $P < 0.01$, $P < 0.001$ and $P < 0.0001$, respectively. Error bars represent the mean ± SD, where n = 3.

Table S1. DNA sequences used in this study

Name	Sequence (5'-3')
Nucleolin H0	AGTCTAGGATTCGGCGTGGGTTAATTTTTTTTTGG TGGTGGTGGTTGTGGTGGTGG
PD-L1 H0	AGTCTAGGATTCGGCGTGGGTTAATTTTTTTTTAC GCTCGGATGCCACTACAGACGGGCCACATCAACT CATTGATAGACAATGCGTCCACTGCCCGTCTCATG GACGTGCTGGTGAC
H1	TTAACCCACGCCGAATCCTAGACTCAAAGTAGTCT AGGATTCGGCGTG
H2	AGTCTAGGATTCGGCGTGGGTTAACACGCCGAAT CCTAGACTACTTTG
T7-crRNA-F	GAAATTAATACGACTCACTATAGGG
T7-crRNA1-R	**TTCGGCGTGGGTTAACACGCC**ATCTACACTTAGT AGAAATTACCCTATAGTGAGTCGTATTAATTTC
T7-crRNA2-R	**ATTCGGCGTGGGTTAACACGCC**ATCTACACTTAG TAGAAATTACCCTATAGTGAGTCGTATTAATTTC
T7-crRNA3-R	**GATTCGGCGTGGGTTAACACGCC**ATCTACACTTA GTAGAAATTACCCTATAGTGAGTCGTATTAATTTC
T7-crRNA4-R	**GGATTCGGCGTGGGTTAACACGCC**ATCTACACTT AGTAGAAATTACCCTATAGTGAGTCGTATTAATTTC
T7-crRNA5-R	**TTAACCCACGCC**ATCTACACTTAGTAGAAATTACC CTATAGTGAGTCGTATTAATTTC
T7-crRNA6-R	**AGTAGTCTAGGATTCGGCGTGT**ATCTACACTTAGT AGAAATTACCCTATAGTGAGTCGTATTAATTTC
T7-crRNA7-R	**AAGTAGTCTAGGATTCGGCGTGT**ATCTACACTTAG TAGAAATTACCCTATAGTGAGTCGTATTAATTTC
T7-crRNA8-R	**AACCCACGCCGAATCCTAGACT**ATCTACACTTAG TAGAAATTACCCTATAGTGAGTCGTATTAATTTC
T7-crRNA9-R	**TAACCCACGCCGAATCCTAGACT**ATCTACACTTA GTAGAAATTACCCTATAGTGAGTCGTATTAATTTC
NS-T7-crRNA10-R	**TTGCTGTATGGTGGGCGTTG**ATCTACACTTAGTAG AAATTACCCTATAGTGAGTCGTATTAATTTC
NS-T7-crRNA11-R	**TCTGAGAATAGTGGTTTGCTGTA**ATCTACACTTAG TAGAAATTACCCTATAGTGAGTCGTATTAATTTC
NS-T7-crRNA12-R	**TTGCTGTATGGTGGGCGTTGAAAGA**ATCTACACT TAGTAGAAATTACCCTATAGTGAGTCGTATTAATTT C
NS-T7-crRNA13-R	**TACCAGTGCGATGCTCAGTGCCGT**ATCTACACTT AGTAGAAATTACCCTATAGTGAGTCGTATTAATTTC
H2-R	CAAAGTAGTCTAGGATTCGGCGTGTTAACCCACG CCGAATCCTAGACT

H2-H2-F	AGTCTAGGATTCGGCGTGGGTTAACACGCCGAATCCTAGACTACTTTGAGTCTAGGATTCGGCGTGGGTTAACACGCCGAATCCTAGACTACTTTG
H2-H2-R	CAAAGTAGTCTAGGATTCGGCGTGTTAACCCACGCCGAATCCTAGACTCAAAGTAGTCTAGGATTCGGCGTGTTAACCCACGCCGAATCCTAGACT
H2-10	AGTCTAGGATTCGGCGTGGGTTAACACGCCGAATCCTA
H2-10-H2-10	AGTCTAGGATTCGGCGTGGGTTAACACGCCGAATCCTAAGTCTAGGATTCGGCGTGGGTTAACACGCCGAATCCTA
Nucleolin-apt-biotin	AGTCTAGGATTCGGCGTGGGTTAATTTTTTTTTGGTGGTGGTGGTTGTGGTGGTGGTGG-biotin
PD-L1-apt-biotin	AGTCTAGGATTCGGCGTGGGTTAATTTTTTTTTACGCTCGGATGCCACTACAGACGGGCCACATCAACTCATTGATAGACAATGCGTCCACTGCCCGTCTCATGGACGTGCTGGTGAC-biotin
Biotin-H1	Biotin-TACCAGTGCGATGCTCAGTGCCGTATCTACACTTAGTAGAAATTACCCTATAGTGAGTCGTATTAATTTC
Biotin-H2	Biotin-TACCAGTGCGATGCTCAGTGCCGTTTCATCTACACTTAGTAGAAATTACCCTATAGTGAGTCGTATTAATTTC
ssDNA-FQ reporter	HEX-TTATT-BHQ1

Yellow highlighted bases indicate 5’ PAM sequences, and the optimal sequence is marked in red; bold sites represent the targeted sequences; NS, non-specific.

Table S2. Information on the NPC serum samples used in Figure 6B/C.

Patient ID	Group	Sex	Age	Pathological stage[a]	EBV-DNA, copy/ml[b]	VCA-IgA[b]	EA-IgA[b]
1	Early	Male	60	T1N0M0, I	0	Missing	Missing
2	Early	Male	34	T2N1M0, II	0	40	10
3	Early	Male	58	T1N0M0, I	0	0	0
4	Early	Male	64	T2N1M0, II	2480	1280	320
5	Early	Female	42	T2N1M0, II	0	0	0
6	Early	Male	46	T2N1M0, II	194	80	0
7	Early	Male	41	T1N1aM0, II	0	0	0
8	Early	Female	26	T2N0M0, II	189	0	40
9	Early	Male	67	T1N0M0, I	0	0	40
10	Early	Female	45	T2N1M0, II	0	160	320
11	Advanced	Male	50	T3N2M0, III	11500	320	1280
12	Advanced	Male	52	T3N2M0, III	1750	80	320
13	Advanced	Male	62	T3N1M0, III	705	160	640
14	Advanced	Male	57	T3N2M0, III	585	40	160
15	Advanced	Female	40	T3N2M0, III	580	0	40
16	Advanced	Male	53	T3N1M0, III	530	160	640
17	Advanced	Male	44	T3N1M0, III	520	160	40
18	Advanced	Male	53	T3N1M0, III	0	160	40
19	Advanced	Male	50	T3N1M0, III	940	640	160
20	Advanced	Male	43	T3N1M0, III	90	160	20

[a] The 8th AJCC staging system;

[b] The data were collected from clinical records.

Table S3. Baseline characteristics of patients treated with anti-PD-1 monoclonal antibody.

Patient ID	Sex	Age	Tumor type [a]	Clinical stage	Endpoint[b]
1	male	31	NPC	TxNxM1IV	SD
2	female	66	lung adenocarcinoma	T2N2M1cIVb	PD
3	female	53	NPC	TxNxM1IV	PR
4	male	39	NPC	TxNxM1IV	PR
5	male	34	NPC	T4N1M1IVb	PR
6	male	66	lung adenocarcinoma	T4N3M1IV	PD
7	male	53	NPC	TxNxM1IV	SD
8	male	57	NPC	TxNxM1IV	PD
9	male	47	NPC	T3N3M0IVb	PR
10	male	48	NPC	TxNxM1IV	PD

[a] NPC, nasopharyngeal carcinoma; [b] Endpoint, SD, stable disease; PR, partial response; PD, progressive disease.

Table S4. EV-derived protein detection method comparison

Detection method	LOD[a]	Dynamic range	Target	Recognition elements	Ref
Thermophoretic aptasensor	3.3 x10^3 particles/μL	10^3-10^7 particles/μL	CD63, PTK7, EpCAM, LZH8, HER2, PSA, CA125, Lib	aptamer	[15]
nPLEX	3,000 counts	10^3-10^7 counts	EpCAM, CD24, CA125, MUC18, EGFR, HER2	antibody	[16]
Dual-signal amplification based on RCA and endonuclease	10^2 particles/μL	10^3-10^5 particles/μL	nucleolin	aptamer	[17]
ExoProfile	21 particles/μL	10-10^6 particles/μL	EGFR, Her2, CA125, EpCAM, CD24, FRα, CD9, CD63	antibody	[18]
ExoPCD-chip	43.9 particles/μL	7.61×10-10^5 particles/μL	CD63	aptamer	[19]
g-C3N4 NSs-CD63 aptamer	13.52 x10^5 particles/μL	0.19×10^5-3.38×10^7 particles/μL	CD63	aptamer	[20]
α-CD9 antibodies electrode	2 x10^2 particles/μL	10^2-10^6 particles/μL	CD9	antibody	[21]
Electrochemical aptasensor based on a Hemin/G‑Quadruplex‑Assisted Signal Amplification	9.54 × 10^2 particles/mL	4.8 × 10^3 to 4.8 × 10^6 particles/mL	MUC1	aptamer	[22]

Strategy					
PLA-RPA-TMA	10^2 particles/mL	10^2-10^8 particles/mL	EGFR, LMP1	antibody	our previous work[2]
apta-HCR-CRISPR	10^2 particles/μL	64-10^6 particles/μL	nucleolin, PD-L1	aptamer	This work

[a] LOD: limit of detection

Table S5. Recovery tests for nucleolin⁺ and PD-L1⁺ EVs in 50% FBS (n = 3)

Group	Added EVs (particles/μL)	PBS[a]	50% FBS[a]	Rate of recovery (%)	RSD[b] (%)
nucleolin$^+$					
1	320	5566	4661	83.7	9.1
2	8000	16065	14951	93.1	9.0
3	10^6	36996	35015	94.6	4.2
PD-L1$^+$					
1	320	4430	5121	115.6	9.0
2	8000	15340	14801	96.5	7.4
3	10^6	37770	38606	102.2	2.8

[a] The fluorescent intensities were adjusted by the background and the blank.

[b] RSD, relative standard deviations. FBS, fetal bovine serum.

References

1. Liao C, Slotkowski RA, Achmedov T, Beisel CL. The Francisella novicida Cas12a is sensitive to the structure downstream of the terminal repeat in CRISPR arrays. RNA Biol. 2019; 16: 404-12.
2. Liu W, Li J, Wu Y, Xing S, Lai Y, Zhang G. Target-induced proximity ligation triggers recombinase polymerase amplification and transcription-mediated amplification to detect tumor-derived exosomes in nasopharyngeal carcinoma with high sensitivity. Biosens Bioelectron. 2018; 102: 204-10.
3. Swarts DC, van der Oost J, Jinek M. Structural Basis for Guide RNA Processing and Seed-Dependent DNA Targeting by CRISPR-Cas12a. Mol Cell. 2017; 66: 221-33 e4.
4. Zhang LK, Huang YC, Xu DD, Yang LX, Qian KC, Chang GZ, *et al.* Biochemical characterization of a thermostable HNH endonuclease from deep-sea thermophilic bacteriophage GVE2. Appl Microbiol Biot. 2016; 100: 8003-12.
5. Yagura T, Schuch AP, Garcia CCM, Rocha CRR, Moreno NC, Angeli JPF, *et al.* Direct participation of DNA in the formation of singlet oxygen and base damage under UVA irradiation. Free Radical Bio Med. 2017; 108: 86-93.
6. Wang YX, Zhang LK, Zhu XY, Li YT, Shi HQ, Oger P, *et al.* Biochemical characterization of a thermostable endonuclease V from the hyperthermophilic euryarchaeon Thermococcus barophilus Ch5. Int J Biol Macromol. 2018; 117: 17-24.
7. Gootenberg JS, Abudayyeh OO, Kellner MJ, Joung J, Collins JJ, Zhang F. Multiplexed and portable nucleic acid detection platform with Cas13, Cas12a, and Csm6. Science. 2018; 360: 439-44.

8. Zhou RX, Li YY, Dong TY, Tang YA, Li F. A sequence-specific plasmonic loop-mediated isothermal amplification assay with orthogonal color readouts enabled by CRISPR Cas12a. Chem Commun. 2020; 56: 3536-8.
9. Roholt OA, Jr., Greenberg DM. Liver arginase. IV. Effect of pH on kinetics of manganese-activated enzyme. Arch Biochem Biophys. 1956; 62: 454-70.
10. Wei H, Therrien C, Blanchard A, Guan SX, Zhu ZY. The Fidelity Index provides a systematic quantitation of star activity of DNA restriction endonucleases. Nucleic Acids Res. 2008; 36.
11. Singh D, Mallon J, Poddar A, Wang Y, Tippana R, Yang O, *et al.* Real-time observation of DNA target interrogation and product release by the RNA-guided endonuclease CRISPR Cpf1 (Cas12a). Proc Natl Acad Sci U S A. 2018; 115: 5444-9.
12. Dai Y, Somoza RA, Wang L, Welter JF, Li Y, Caplan AI, *et al.* Exploring the Trans-Cleavage Activity of CRISPR-Cas12a (cpf1) for the Development of a Universal Electrochemical Biosensor. Angew Chem Int Ed Engl. 2019; 58: 17399-405.
13. Chen Y, Yang S, Peng S, Li W, Wu F, Yao Q, *et al.* N1-Methyladenosine detection with CRISPR-Cas13a/C2c2. Chem Sci. 2019; 10: 2975-9.
14. Dirks RM, Pierce NA. Triggered amplification by hybridization chain reaction. Proc Natl Acad Sci U S A. 2004; 101: 15275-8.
15. Liu C, Zhao J, Tian F, Cai L, Zhang W, Feng Q, *et al.* Low-cost thermophoretic profiling of extracellular-vesicle surface proteins for the early detection and classification of cancers. Nat Biomed Eng. 2019; 3: 183-93.
16. Im H, Shao H, Park YI, Peterson VM, Castro CM, Weissleder R, *et al.* Label-free detection and molecular profiling of exosomes with a nano-plasmonic sensor. Nat Biotechnol. 2014; 32: 490-5.
17. Huang L, Wang DB, Singh N, Yang F, Gu N, Zhang XE. A dual-signal amplification platform for sensitive fluorescence biosensing of leukemia-derived exosomes. Nanoscale. 2018; 10: 20289-95.
18. Zhang P, Zhou X, Zeng Y. Multiplexed immunophenotyping of circulating exosomes on nano-engineered ExoProfile chip towards early diagnosis of cancer. Chem Sci. 2019; 10: 5495-504.
19. Xu H, Liao C, Zuo P, Liu Z, Ye BC. Magnetic-Based Microfluidic Device for On-Chip Isolation and Detection of Tumor-Derived Exosomes. Anal Chem. 2018; 90: 13451-8.
20. Wang YM, Liu JW, Adkins GB, Shen W, Trinh MP, Duan LY, *et al.* Enhancement of the Intrinsic Peroxidase-Like Activity of Graphitic Carbon Nitride Nanosheets by ssDNAs and Its Application for Detection of Exosomes. Anal Chem. 2017; 89: 12327-33.
21. Doldan X, Fagundez P, Cayota A, Laiz J, Tosar JP. Electrochemical Sandwich Immunosensor for Determination of Exosomes Based on Surface Marker-Mediated Signal Amplification. Anal Chem. 2016; 88: 10466-73.
22. Huang R, He L, Xia Y, Xu H, Liu C, Xie H, *et al.* A Sensitive Aptasensor Based on a Hemin/G-Quadruplex-Assisted Signal Amplification Strategy for Electrochemical Detection of Gastric Cancer Exosomes. Small. 2019; 15: e1900735.
